# Why emotions need to labor—Influencing factors and dilemmas in the emotional labor of Chinese English teachers teaching online

**DOI:** 10.3389/fpsyg.2022.981500

**Published:** 2022-09-29

**Authors:** Huaidong Wang, Nuankun Song

**Affiliations:** ^1^School of Journalism and Communication, Renmin University of China, Beijing, China; ^2^College of Teacher Education, Capital Normal University, Beijing, China

**Keywords:** emotional labor, English teacher, influencing factors, dilemmas, online teaching

## Abstract

During the COVID-19, online teaching has become a popular way of teaching in the world. Previous research on English language teachers’ emotional labor has not focused on the changes brought about by online teaching. Unlike the traditional physical teaching space, the emotional labor of English teachers teaching online changes with the daily use of online technological conditions. Therefore, this paper aims to investigate the factors influencing teachers’ emotional labor in online teaching and the emotional labor dilemmas. We took interviews with a group of English teachers in China (T1, T2, … T20) and found that their online teaching emotions were influenced by three main factors: the degree of adaptation to online teaching technology, the invisibility of the online teaching space, and the peripheral environment of the space in which the teachers were located. In addition, this study observed the online classrooms of the interviewees and found that the first two influences are at a priority level for Chinese English teachers, while whether the third factor can bring positive or negative emotions is often influenced by the nature of the emotions brought by the first two factors. In order to find a balance between norms and emotions, English teachers are often accompanied by emotional labor in their work. However, they face many dilemmas of choice and behavior during the emotional labor of teaching online: emotional rule dilemma, emotional expression dilemma and professional identity dilemma. The emergence of these dilemmas is directly related to the influencing factors. In conclusion, this article uses normative and emotional labor theories to explore how norms affect Chinese English teachers’ emotions when teaching online as a response to the question of why emotions need to be labored. Ultimately, this study offers some useful suggestions for language teachers’ emotional labor dilemmas.

## Introduction

Emotions have been generally ignored or avoided in educational research (Sutton and Wheatley, 2003), due to the traditional perception of education as a rational endeavor (Ashforth and Humphrey, 1995) and the norms of the profession that require teachers to control their emotions (Winograd, 2003). This stems from the fact that in the philosophical system reason and emotion are placed in a dichotomous relationship (Yin, 2008). The emotions have always lurked in the background—often as a threat to reason (Solomon, 1993). However, as a natural person, human emotions accompany human daily activities.

The theory of natural evolution holds that emotions are inherent to human physiological patterns and are adaptive responses that have evolved (Panksepp, 2005). Along with the attention of many researchers to the phenomenon of emotions in teacher practice, educational research has gradually taken an emotional turn (Beatty, 2000; Hargreaves, 2000), and teacher emotional labor, including the emotional labor of English teachers, has become a hot topic of research. In the context of the COVID-19, emotions are magnified in all social professions, as well as in the teaching profession. Like a magnifying glass, the COVID-19 has made teachers’ emotional labor an everyday occurrence, due in large part to a dramatic change in teaching methods: Offline classes have become online (Adedoyin and Soykan, 2020; Ortiz, 2020). Online teaching has broken the original teaching model, online teaching emotional labor has changed dramatically. In the offline classroom, students and teachers are in the same physical space most of the time, and teachers are visible in all their actions, which makes them hide their negative emotions in order to conform to professional norms, establish a teacher’s self-image, and better achieve teaching effectiveness. In the online teaching process, teachers and students are completely separated into two spaces, which makes teachers’ behaviors and emotions invisible to students most of the time, the emotional labor of teachers teaching online becomes a visible factor in the education industry compared to the need to hide emotions from students offline. This is related to the important role of Information and Communication Technologies (ICTs) in online teaching (Tartavulea et al., 2020). It reconfigures various aspects of everyday life and of processes of contemporary subjectivation and socialization (Lasén, 2010). Emotional labor and management characterize a big part of the way people experience affectivity, and both are increasingly mediated by ICTs (Lasén, 2013). Studies like this view also point out that the digital realm has its own peculiarities, which come from its electronic nature, and that in turn affect the emotional dimension of the person (Serrano-Puche, 2016). In this daily practice of human-computer interaction, it has also led to issues such as emotional imbalance, depression, reduction in interpersonal communication etc. (Pachalag and Malhotra, 2017), because (a) the teacher’s emotion expression was higher as a result of the teacher’s use of video in the online course (Borup et al., 2012), (b) shorter time span (30–90 min) of emotional arousal in a more restricted environment (Lehman et al., 2012). We can see a common phenomenon around us, there are many teachers who are constantly flipping through their phones to check their schedules in order to prepare in advance for what they are going to do next, seemingly under the constant “tyranny” of time. At the same time, online teaching is restricted by more conditions, such as the use of technology, teacher-student interaction, etc. (Noor et al., 2020). That is, the more emotionally intense the teachers’ labor will be in online teaching.

There are several norms in the teaching profession to curb teachers’ emotions so that their emotions do not interfere with their teaching. As emphasized by normative theory, specific objects or events generate their own norms by retrieval of similar experiences stored in memory or by construction of counterfactual alternatives (Kahneman and Miller, 1986). This means that norms are constructed from similar experiences of “how things have been done before” or through the opposite of things—“what not to do.” Some papers also noted norms, which were used as a basis for teachers to choose the right strategies for emotional labor (Yin et al., 2017). Professional norms are also seen as factors that control and dominate teacher performance (Ghanizadeh and Royaei, 2015). Teachers should try to behave in compliance with formal and informal norms as professionals (Yilmaz et al., 2015), emotion management or emotional labor occurs when accommodating these norms (Wharton, 2009). As can be seen, previous studies have emphasized that teacher professional norms determine teacher emotions. Our study, however, intends to build on this foundation by examining how professional norms affect the emotional labor of Chinese English teachers teaching online, further reflecting on normative theory to some extent. Meanwhile, we need to note that different identities are accompanied by different emotions (Mercer, 2014), and the profession of teaching refers to social identities, while the teacher as self is outside the norms of the teacher. One study noted that the teachers’ self is indistinguishable from the professional role (Jeffrey and Woods, 1996). We consider that this is a question of the boundary between the self and the role, a boundary that is difficult to distinguish clearly because the subject of the occurrence of emotions remains unchanged, and the professional role of the teacher requires hiding some negative emotions, but it does not mean that negative emotions disappear. Instead, according to our daily observation, they are transferred to the world of the self during the non-role identity time. Today, we learned through interviews that teachers are increasingly inseparable between online and offline, in addition to online teaching, teachers need to use computer to prepare teaching materials, course slides, and correct homework. Language carries strong emotional properties and is one of the ways to express emotions, so in countries where English is a foreign language, teaching English is different from all other subjects, and both teacher transmission and student learning need to switch to another cultural model different from the native one. Therefore, emotional research among foreign language teachers also continues to emerge (De Costa et al., 2018).

Emotional labor was originally defined as an employee changing how she feels, or what feelings she shows, in order to interact with customers or clients in an effective way (Hochschild, 1983). The focus of the conception is on the management, or modification, of emotions as part of the work role, managing emotions for a wage has been termed emotional labor. But in China, teaching is more than an ordinary job with the first purpose of earning money, it is considered a profession with a public interest, and in the view of teachers the first purpose of their work is to bring people knowledge and scientific methods of perceiving the world. Therefore, in the emotional labor of Chinese English teachers, they pay much attention to professional norms and teachers’ social responsibility. Online teaching has reshaped the scenes and contexts of English teachers’ emotional labor, and the factors influencing emotional labor have changed. While emotional labor in the real space is more reflected in interpersonal interactions between teachers and students (Bao et al., 2022), online teaching adds computer-mediated variables, which brings new changes to teachers’ emotional labor. Excessive emotional labor in teaching online can cause burnout (Pyhältö et al., 2011), thus creating an emotional labor dilemma for teachers. Previous studies on English teachers’ emotional labor dilemmas (Jacob, 2007; Kersaint et al., 2007; Guan, 2020) have focused on what dilemmas English teachers face after emotional labor, without considering the many choice dilemmas that English teachers also face when emotional labor occurs, so this study intends to examine the dilemmas that English teachers face when emotional labor occurs in China.

In summary, this paper analyzes the factors influencing the emotional labor of Chinese English teachers teaching online and the dilemmas encountered in the process of emotional labor. The research gap of this thesis lies in the fact that previous discussions of teachers’ emotional labor and English teachers’ emotional labor in terms of influencing factors are based on the reality of teaching in physical space, a teaching model in which emotional labor arises mainly from interpersonal relationships between teachers and students, teachers and administrators, however, our study focuses on English teachers teaching online, whose emotional labor arises primarily from computer-mediated human-computer relationships that have not been discussed before. Previous discussions of English teachers’ emotional labor dilemmas have focused on the effects on English teachers themselves following their emotional labor, but our study focuses on another aspect, English teachers teaching online also generated dilemmas in terms of emotional choices during their emotional labor after being stimulated by a certain event. We do not repeat the study of what kind of dilemmas English teachers face after emotional labor, and turn attention to what kind of emotional difficulties they face when their emotional labor occurs. Thus, the research questions in this paper are significantly heterogeneous from previous studies. This study aims to explore the factors influencing Chinese English teachers’ emotional labor in online teaching, to add a new online context to the theory of emotional labor, and to draw more researchers’ attention to and further research on English teachers’ emotional labor online. In addition, this paper analyzes Chinese English teachers’ emotional labor dilemmas in online teaching so that more people can recognize that they not only generate dilemmas after emotional labor, but also emotional dilemmas when emotional labor occurs. We also provide some thoughts and suggestions to solve these dilemmas of English teachers.

## Literature review

### Teachers’ emotional labor

Hochschild used the term emotion management to describe how workers control, manage their emotions to make sure that they are expressed in a way that is consistent with social norms or expectations (Hochschild, 1983). This concept was later developed into emotional labor and used by many researchers as a classical theory to explain human emotions (Hesmondhalgh and Baker, 2008; Theodosius, 2008; Badolamenti et al., 2017). Emotions in teaching have gone through a process from masking to de-masking, and emotions are now considered to be intimately involved in virtually every aspect of the teaching process (Schutz and Lanehart, 2002). Some emotions such as anxiety, anger, and disappointment become emotional labor when teachers engage in efforts to modify and control negative emotions for the purpose of expressing only those emotions that are socially acceptable (Isenbarger and Zembylas, 2006). In addition to these unacceptable negativities in social norms, in the view of managers, the more volatile, passionate emotions (which are also the less easily managed ones), like joy, excitement, frustration and anger are kept off the educational agenda in favor of ones that encourage trust, support, openness, involvement, commitment to teamwork and willingness to experiment (Hargreaves, 1998). The research on emotional labor of teachers has focused on three questions: What is the impact of emotional labor? How can teachers conduct strategies for emotional labor? What are the factors that influence teachers’ emotional labor?

There is some research that suggests that emotional labor in teaching can have a positive impact (Hebson et al., 2007; Mack, 2008; Kimura, 2010; Tsang, 2011; Lyndon et al., 2021). They perceived the impact of teaching emotional labor as positive, including teachers’ satisfaction with their jobs, self-efficacy, and other aspects. However, some studies have concluded that the effects of emotional labor are negative (Cukur, 2009; Yin, 2009; Hülsheger et al., 2010; Martínez et al., 2020). They argue that the impact of emotional labor in teaching is negative, included issues such as emotional burnout, professional identity. One study has critiqued the idea of negative impact, arguing that it would be caused by the misconceptions and misinterpretations of the concept of emotional labor (Tsang, 2011). This criticism denies that emotional labor has negative consequences. We should not directly characterize the impact of emotional labor as having only one positive or negative dimension, but we need to consider the specific context in which emotional labor occurs, and the impact of emotional labor possesses both positive and negative aspects. Teachers enjoying positive interactions with students and colleagues, recognition from school, family and public, but experiencing negative emotions in relation to unfair treatment, competition among colleagues, imbalance of work lives, and pressure from society, policy, and educational change (Chen, 2016).

The impact of teachers’ emotional labor can sometimes go beyond professional norms, that is why teachers have emerged with emotional labor strategies, a topic that has also been examined. It is important for teachers to find appropriate strategies for emotional labor, because managing emotions has been seen as essential in effective teaching (Fried, 2001; Taxer and Frenzel, 2015), so researchers have begun to focus on teachers’ emotional labor strategies, exploring how teachers manage their emotions in the teaching process to conform to social or managerial norms and to promote teaching effectiveness at the same time. Teachers are perceived by the general public as a profession that faces multiple pressures such as policy agendas and requirements (Day, 2014), teaching stress (Harmsen et al., 2019). So, they use strategies to maintain their commitment and well-being (Beltman and Poulton, 2019), follow the caring ethics (Noddings, 1996) by suppressing emotions (Gross, 2002) and using relaxation techniques (Webster and Hadwin, 2015).

The factors influencing teachers’ emotional labor are to be divided into two dimensions. On a macro level, emotional labor is affected by the interactive situation (Dunkel and Weihrich, 2013), social culture (Tösten and Sahin, 2017), the characteristics of the emotional labor and interactive objects (Dunkel and Weihrich, 2013). On a micro level, teachers’ emotional labor is influenced by a variety of factors. (1) Personality Characteristics, such as big five personality traits can affect emotional labor strategy (Basim et al., 2013). (2) Emotional Intelligence, Emotional intelligence significantly moderates the impact of emotional job demands on surface acting and expression of naturally felt emotion but not deep acting (Yin, 2015). (3) Psychological Capital, include self-efficacy, optimism, hope, and resilience (Vanno et al., 2014). A positive psychological capital can diminish the negative effects of emotional labor (Tamer, 2015; Tosten and Toprak, 2017).

### English language teachers’ emotional labor

As for emotional labor of English language teachers, some papers mostly focus on describing and comparing the experience of emotional labor in various countries: Japan, Pakistan, Iranian, Korea (Kim and Kim, 2018; King and Ng, 2018; Fathi and Derakhshan, 2019; Taylor, 2020; Greenier et al., 2021).

Several studies have analyzed the factors influencing English teachers’ emotional labor. Emotional labor has been associated with job dissatisfaction, health symptoms and emotional exhaustion (Schutz and Lee, 2014). Top-down institutional policies that may conflict with English language teachers’ training and/or pedagogical preferences thereby producing emotion labor (Benesch, 2020; Nazari and Karimpour, 2022). A struggle over which emotions are suitable may therefore be present (Benesch, 2017), such as they spot plagiarized portions of students’ texts (Benesch, 2018). English teachers are asked to use positive emotions in the classroom, both in regulating their own emotions and those of their students. But emotional energy they expended on eff orts to regulate these positive emotional displays in class can lead to emotional labor (Acheson et al., 2016), because teachers suppress their own negative emotions while attending to those of their students (Gkonou and Miller, 2019). Others believe that emotional labor of English teachers largely attributed to the subject’s value-laden content, the stresses of grading student essays, the performance pressures of high-stakes testing, and the need for culturally responsive pedagogies (Loh and Liew, 2016). Some researchers have noted the emotional labor of non-native English teachers, if the teachers live in a foreign country and need to adapt to the context linguistically, socially, and culturally (Cowie, 2011). Educational background, competence in the local language, and supportive discourses at work led to different emotional labor in their lives (Kocabaş-Gedik and Ortaçtepe Hart, 2021).

However, the above factors influencing teachers’ emotional labor are based on teaching in the traditional physical space, online teaching has become a new context and characteristics for this topic. The impact factors, etc., need to be combined with the specific characteristics of the work to do further research (Ye and Chen, 2015). We find that previous researches related to English teachers’ emotional labor has not focused too much on the online teaching field for the time being. But emotions in online learning have gained more attention (Artino, 2012). Meanwhile, there are articles that focus on online teaching during the COVID-19, but are not related to the emotional labor of teachers, including English teachers. They focused on advising teachers on how to embrace the digital classroom, how to adopt teaching strategies, etc. (Gewin, 2020; Mahmood, 2021). In fact, online teaching as a result of the COVID-19 has clearly changed the way teachers’ emotional labor and the impact of emotional labor. This is where our research gap lies. The emotional labor of online teaching involves the relationship between networks and emotions. Digital devices facilitate some exchanges, activities, and modes of control, they contribute to the eliciting, expression, communication, and management of affects and emotions (Lasén, 2013). In the digital environment, where there is no corporeality that accompanies physical relationships, and the communication between participants is not necessarily synchronous (Serrano-Puche, 2016). So, what changes can these technological features of online teaching bring to the emotional labor of English teachers? In other words, what are the factors of emotional labor for English teachers teaching online?

As for the dilemma of English teachers’ emotional labor, dilemmas refer to “a situation in which the participants are required to manage competing alternatives.” (Settelmaier et al., 2004). Previous research has focused on the negative effects that come after the emotional labor of English teachers, which is discussed as a result of emotional labor. A growing number of researchers suggest that teachers leave the profession at high rates during the first few years of teaching (Kersaint et al., 2007). English language teachers are even higher for this number (Jacob, 2007). The mental burnout of English teachers was also examined. Mental burnout mainly arises from the contradictions between expectation and reality, work and family, or teachers and students, as well as pay and return (Guan, 2020). What these studies illustrate are the negative effects of emotional labor for English teachers, and we do not deny that this is a dilemma that English teachers face after emotional labor, such as facing the dilemma of choosing between leaving and professional commitment, or there are others. But emotional labor is a process, and English teachers’ emotional labor has a time span and a choice process. In our study, we found that they face normative and emotional conflicts during this time span, and we wanted to explore what kind of dilemmas they face during this time span. However, previous papers have mainly focused on the dilemmas of English teachers after emotional labor as a result, and what kind of dilemmas English teachers face during emotional labor has not been given much attention, which is where our research gap lies. We all know that online teaching has brought fundamental changes to the emotional labor process, what kind of dilemma English teachers face during the emotional labor of teaching online is a new question worth exploring. As we can see, teachers’ emotional labor includes English teachers’ emotional labor, previous studies have not explored emotional labor in online teaching, the dilemmas are discussed as a result of emotional labor afterward. We did not want to repeat the study of dilemmas as a result of emotional labor afterward, but rather to examine the dilemmas that Chinese English teachers face when emotional labor occurs. Based on previous studies, we want to further examine the factors that influence Chinese English teachers’ emotional labor in online teaching and the emotional labor dilemmas of online teaching.

## Methodology

### Participants

In this paper, a research method of interviews and online classroom observations was used. Firstly, in-depth interviews were conducted with 20 Chinese English teachers (T1…T20; Table 1). Before the interviews, the concept of emotional labor was explained in order to confirm their perceptions of the research questions and to confirm that they were in this labor process. Secondly, we observed the interviewees in their online classrooms in order to get a more accurate picture of the teachers’ emotional changes in their online teaching, to avoid situations in which they did not think or did not speak completely during the interview. We briefly recorded the nodes of their emotional changes during the observation process, and what happened before and after the emotional changes. If questions that warranted additional interviews arose during the observation of the online classroom, we also re-interviewed the teachers after the course to ensure that the normal classroom order was not disrupted. Considering the impact of gender differences in emotional labor on the study results, we included nine male teachers and 11 female teachers in the interviews. Our interviews were conducted online due to the COVID-19.

**Table 1:** Information of interviewees.

Number	Province	Gender	Age	Teaching experience (year)
T1	Beijing	Male	29	5
T2	Beijing	Female	32	6
T3	Henan	Female	36	10
T4	Henan	Female	37	12
T5	Henan	Male	27	4
T6	Gansu	Female	30	5
T7	Gansu	Male	50	22
T8	Yunnan	Male	28	4
T9	Qinghai	Female	27	4
T10	Ningxia	Female	45	19
T11	Shanxi	Male	30	4
T12	Hubei	Male	35	7
T13	Sichuan	Female	50	26
T14	Fujian	Male	47	24
T15	Hunan	Female	27	2
T16	Hebei	Female	29	5
T17	Zhejiang	Male	34	6
T18	Shandong	Female	49	25
T19	Shaanxi	Female	35	10
T20	Guangdong	Male	29	5

### Instruments

The emotional labor of operationalization is defined as “showing real emotions, displaying pretended emotions and to suppress own mood and emotions.” (Glomb and Tews, 2004; Zhang et al., 2006). We used this operationalization as the basis for our interview outline design. Because we made some preparations before the formal interview by explaining the concept of emotional labor to each interviewee and confirming that he or she was in the process of emotional labor, some questions on basic concepts were omitted from the interview outline. We mainly interviewed the following questions:

1. How much time do you spend teaching online each day? Including all your teaching-related work such as lesson preparation, teaching, and homework correction.
2. What do you think are the main difficulties faced in online teaching?
3. What do you think are the factors that influence your emotional labor in online teaching?
4. What differences does online teaching bring compared to teaching in the real space?
5. What effort did you need to put into adapting to these differences? Were there any negative emotions during the adaptation process?
6. Does online teaching have an impact on the expression of your true emotions? Do you display faked emotions? Or suppress your emotions?
7. What are the main dilemmas you face in online teaching when you feel like doing emotional labor, or seeking an appropriate emotion?
8. How is the dilemma faced by the emotional labor process in teaching in real space different from online teaching?

### Analysis of data

To answer the research question, we analyzed a total of 20 transcripts and some records of classroom observations. We coded and categorized the specific conditions of the various types of emotional labor of the teachers in the interviews, aided by the observation of the online teaching classroom, trying to analyze what factors were affecting the emotional labor of online teaching and to do an in-depth analysis of the emotional labor dilemmas they faced.

The basic principles of 20 transcription codes are as follows: We attributed the difficulties they encountered in using the online teaching software such as not being able to find the corresponding function buttons, not knowing how to operate them to achieve good teaching results, and the lag caused by the course videos, which triggered emotional labor, to the first factor. We attributed the problem of online video visibility to the second factor. For example, teachers felt some concern when they could not see students’ serious lessons in the screen, and teachers also worried that they might have some inappropriate emotions and facial expressions seen by students in the screen and formed a psychological burden. The third factor was the emotional labor caused by the noise of renovation and pets in the class.

We recorded the teachers’ emotional change nodes in our classroom observation, such as the teachers’ expression change and tone change before and after the disruption because of the disruptive person among the students disrupting the normal classroom order; or the teachers’ suppression of their emotions by pausing; in addition, the emotional changes that occurred because of the situations corresponding to the three influencing factors were also included in our records.

In order to ensure the reliability of the coding, we coded and exchanged checks on the emotional changes and categorizations used in the research process, and any disagreements were discussed and finally agreed upon. Finally, we came up with three factors influencing Chinese English teachers’ online teaching emotional labor and three emotional labor dilemmas they face.

## Results

### Influencing factors of Chinese English teacher’s emotional labor teaching online

#### The degree of adaptation to online teaching technology

After interviewing 20 English teachers in China, the online teaching software they use daily are Tencent Meeting and Zoom. Technical problems have been plaguing them in the use of these tools. Especially before the class, it always takes some time to test the network, camera, microphone, etc., in case the students cannot see the teacher and hear the voice. 13 of them (T1, T2, T5, T6, T8, T9, T11, T12, T15, T16, T17, T19, T20) thought that they needed at least 5 min to complete this work, and 7 of them (T3, T4, T7, T10, T13, T14, T18) needed 10 min to prepare. Over time, the software has also been updated with new features, which has caused some of the original buttons to change, and teachers have had to continually adapt to the software.

“When I first started teaching online, it was the most difficult time for me. I usually had to ask my daughter to help me adjust the online teaching software before the class. Especially since the software was frequently updated and I had just learned the old version and a new one came out, so I was always adapting to online teaching technology, which makes me distressed and angry” (T7).

This breaks the original teaching procedure and requires more time from the teacher. In the real space teachers, the computer used by the teacher is already set up and does not require additional work from the teacher. In addition, teachers are also concerned about whether there will be network lag or other accidents during the lesson that will cause students to have a bad experience, teachers’ attention is also more distracted than usual. In comparison, unpleasant emotions emerge. However, teachers have professional norms, the teaching profession requires teachers to hide some negative emotions. “Of course, we cannot express too negative emotions, our expressions will be caught by the students, which will affect their learning” (T5, T12, T17). The teachers chose to hide their emotions in order not to let them interfere with their normal lectures. We need to note that hiding does not mean disappearing, because emotions not only have external manifestations, but also can cause certain internal reactions in the body, such as rapid heartbeat, muscle tension, etc. When teachers’ negative emotions are hidden through emotional labor, they tend to talk to their families after the session about how they felt at the time. “Without a doubt, my husband is my first choice to talk about my unpleasant emotions that arise in the online classroom” (T3). Hiding is only to follow professional norms. Emotional labor then becomes a game and negotiation between professional norms and emotions. It should be noted that this does not mean that they think the norms are wrong, and they also think it is right that emotions do not interfere with teaching, but they have no way to make the negative emotions disappear. Hiding emotions cannot hide the physical unpleasantness that emotions bring. There are studies shows, those who report regularly having to display emotions at work that conflict with their own feelings is more likely to experience emotional exhaustion (Levy, 2018), so this is the first factor of emotional labor.

#### The invisibility of the online teaching space

Our interviewees have all experienced situations where students are online but not in class. Teachers often saw a student online on the computer screen, but no one responded when their name was called.

“This is often the case, and it makes us very frustrated. If we are in a real space, we can walk up and remind or warn students who aren’t paying attention, but there’s no way to do that with online teaching” (T1, T8, T19).

The invisibility of the online space prevents teachers from seeing what students are always doing, which is a major concern for teachers in class. Whereas in the offline space, students listen attentively under the teacher’s watchful eye, the online space requires teachers to find more ways and energy to ensure that students are paying attention to the lesson. However, our interviewees said this was difficult to achieve.

“Punishment does not work, and our punitive measures do not inherently do much good because we need to be sure of the scale of punishment, and punishments that are too severe aren’t allowed. Students will also state reasons that seem to fit the norm” (T2, T11).

It is a fundamental part of the teacher’s code to make sure that every student is paying attention, and when teachers do everything that they can to achieve this goal but failed finally, they become frustrated and doubt their own teaching abilities.

“When I feel frustrated, angry, etc., it should not affect the effectiveness of teaching, we need to suppress and transform our negative emotions and mobilize positive emotions to continue to do our work” (T6).

Teachers cannot give up trying because their status as teachers gives them a strong sense of responsibility and they need to work hard to make every student understand the content of their lessons, which is their professional commitment. Especially as English teachers, the language they teach is not their own or their students’ native language, this can cause anxiety that different from other subject, and once students miss the course, the social and cultural contexts that the language corresponds to cannot be understood for Chinese students, which can produce bad teaching results. In addition, the invisibility involves the construction of teacher-student relationships. Our observations of the online classroom found that effective teacher–student interaction is an essential element of an ideal classroom, which promotes the opportunity for expanded learning and stimulates the connections between students and teacher. We saw that when the teacher’s lectures are becoming more active, students are responding to the teacher more frequently. “When we show kindness and enthusiasm, our students also feel closer to each other and they show more courage to answer questions” (T10, T14, T16). According to the usual network theory, the interaction between the teacher and the students in the online classroom is synchronized, but the fact is that when the teacher does not ask a question by name, the student’s network account is online but the student can be offline. What is important is that the teacher’s communication with students creates some problems because of invisibility. When the students are not visible to the teacher, the teacher cannot see the students’ emotional reactions to him or her, and considering the effectiveness and satisfaction of teaching, the teacher wants to see the students’ reactions in order to judge the effectiveness of his or her lectures.

“Now I speak to the computer and before to the students, there is still a big difference. I cannot see the students’ reaction, I do not know whether they understand or not, so I am constantly worried during the lesson” (T8).

When the teacher is not visible to the students, the teacher is in a prior position of power in the educational process and the students tend to imagine and speculate about the teacher’s emotions during the lesson. We saw an interesting phenomenon in the classroom observation: The teacher asked a question to a student, but he answered wrongly, so he typed “Is the teacher angry?” in the chat box, but he withdrew it after a few seconds. “I have encountered several times, they actually wanted to send to their close classmates, but forgot to select the sending range” (T5, T19). But in both above cases, there are often errors in understanding emotions. Thus, the problem of invisibility also becomes the second factor of emotional labor.

#### The peripheral environment of the space in which the teachers were located

We found through participatory observations of online classrooms that the peripheral environment in which teachers are placed affects their emotional labor. Some studies have examined the impact of teachers’ work environment on emotion (Yin et al., 2016; Lyndon et al., 2021), but the environment involved refers to the relational environment between people rather than the environment of the physical space. We consider that the teacher’s peripheral environment includes all the noises generated in the home or office, such as the barking of pets, noise from renovations, weather-related storm sounds, which can affect the teacher’s mood. Because keeping the classroom quiet is part of the teacher’s norm. When these noises in the environment suddenly interrupt the classroom, teachers feel negative emotions and want the sounds to go away. But when this sudden situation occurs, teachers’ emotional reactions vary somewhat depending on the situation, which is related to the two previous factors. We found that when teachers were skilled at using software to quickly avoid these sounds, such as quickly finding the sound button to turn off the microphone so that students could not hear the noise in their environment, they had less negative emotional reactions to the noise.

“For the teaching profession, the classroom has a unique meaning to us, and we do not want to be disrupted or disturbed by anything, but online teaching cannot avoid some unexpected situations, and if we can handle them in time, we will also be positive about short bad moments like this” (T4, T15, T16).

But when the teacher does not know how to avoid the noise, the longer the noise continues, the angrier the teacher becomes and the more visible it is on their face. In our classroom observation notes, we found that five teachers (T4, T7, T10, T13, T18) were unable to solve the problem in time when they encountered the continuous noise of renovation interrupting the class, and eventually they had to tell the students to end the class first, and the teachers’ facial expressions turned from normal to unpleasant. Just like a stage play, there is a mistake in front of the audience (Goffman, 1959). Furthermore, invisibility has something to do with it. When sudden sounds intrude, if the teacher turns off the camera (in a state that makes the students invisible), the less embarrassed the teacher will feel and the less negative emotions will be felt. When an unexpected situation occurs with the teacher’s camera on (in a state that makes the students visible), the more anxious the teacher will be if he or she cannot solve it.

“If I turn off the camera, which seems to allow me to avoid some embarrassment, but open the camera, the presence of so many students I feel particularly embarrassed and must hide this embarrassment” (T7, T18).

Therefore, we believe that the more adaptive the teacher’s online technology is, the less visible the teacher is to the students and the more positive the emotion of dealing with the peripheral environment, and vice versa.

### The emotional labor dilemma of Chinese English teacher teaching online

The emotional labor dilemma arises in relation to the influencing factors. We need to clarify the logical relationship between the influencing factors and the emotional labor dilemma, because the factors that influence emotional labor in online teaching produce a difference, the emotional labor dilemma appears to change accordingly.

#### Emotional rule dilemma

This relates to the first influencing factor. Central to all theories of emotional labor is the idea that individuals follow emotional display rules that specify the appropriate expression of emotions on the job (Diefendorff and Richard, 2003). The difference between English teachers and teachers of other subjects is that English teachers have slightly different perceptions of the content of emotional rules because the language they teach is not their native language or Chinese, and they are influenced by the culture that corresponds to the language. In the original teaching, English teachers had some differences in the way they understood their relationships with school leaders, other teachers, and students. The rules that come with the use of technology in online teaching trigger more emotional rules. Our interviews showed that English teachers cared more about their feelings, but the more they cared also indicated the more emotional labor they needed to do and the more intense it was, given the professional norms.

“Some teachers around me choose to obey rules and norms to a great extent, but this is different from my idea, I think I need to pay attention to my own feelings and emotions, but this often conflicts with school rules or professional norms” (T8).

Some researchers argue that emotional arousal is limited by rules that force people to perform emotional labor to suppress inappropriate emotions (Susan, 1979). “One thing we need to understand is that there are so many emotional rules in online teaching that it has revolutionized the way people communicate with each other” (T14). But in the view of English teachers, neither emotions nor norms are wrong, and they have difficulty making a negotiation between the two in the course of their work.

#### Emotional expression dilemma

This relates to the second influencing factor. Ambivalence is seen as an important mediator in the link between emotional styles and psychological and physical well-being (King and Emmons, 1990). Emotional expression dilemmas arise primarily from ambivalence in classroom management. In English teachers’ online classrooms, they are often accustomed to ask students questions at any time to observe whether their pronunciation is accurate or not. However, in the online space, teachers find that when some students do not respond when they are called to speak, teachers realize that there is no one behind the screen. All our interviewees reported having been in this situation. Especially when students are not supervised by their parents, they are likely to go to sleep, play something else, and be online with just a study account. “This happens often, so students need to be asked questions at all times during class” (T12, T19). In addition, when teachers are lecturing, there are students who often forget to turn off their microphones, and when teachers are frequently disturbed by other loud noises, which means classroom discipline is not taken seriously and their teaching effectiveness suffers, teachers are faced with the dilemma of expressing emotions: Should they express emotional words or expressions or not? If the teacher expresses emotions, it may cause psychological stress and lead to boredom, but if not, the students may never listen carefully to the lesson. “This dilemma is basically the norm for online teaching, and we need to be very careful about tone and expression” (T13, T16). It is difficult for them to choose, and even if they do, it is difficult to grasp the scale of expression because the teacher does not know how well the students perceive and understand emotions.

“Teaching in the real space, we can know the students’ psychological state at any time. But teaching online, it is difficult to know the students’ psychological state when the course is over, we must consider the matter of students’ mental health” (T10).

Therefore, we regard whether emotions should be expressed or not as one of the dilemmas of English teachers’ emotional labor.

#### Professional identity dilemma

This relates to the third influencing factor. Some studies have pointed out, the mental health of the teacher affects the emotional atmosphere of the classroom which in turn impacts students’ experience of education (Vesely et al., 2013). But the emotional burnout of teachers in online teaching also needs to consider the effects of Internet use on teachers. The digital realm has its own peculiarities, which come from its electronic nature, and that in turn affect the emotional dimension of the person (Serrano-Puche, 2016). Our interviewees, especially older teachers (T7, T10, T13, T14, T18), were sedentary in front of the computer, causing soreness in their eyes, back, and other body parts. A range of teaching activities, such as lesson preparation, lecturing, and homework review, all take place online, and teachers need to meet deadlines and requirements in front of the computer to complete their teaching tasks.

“Completing teaching tasks is a basic requirement for us as teachers, but the process is a little more difficult in the context of online teaching than it was in the real space. The process of many things is complicated by the fact that they are done online” (T3, T17).

In addition, compared to the offline physical space, it is difficult for teachers to effectively remind students to take their lessons seriously online and to interact with them in a positive way. At the same time, online teaching is less efficient than offline teaching, but the requirement of teaching assessment still exists. “Sometimes the teaching requirements are even stricter than before, because in the administrators’ opinion, they cannot supervise us at all times like before” (T1, T4). A variety of factors contribute to emotional labor that can cause burnout and further professional identity dilemmas. As our interviewees (T7, T20) said, “sometimes I think about the boundaries of my identity, what I should do and what I should not do, and if it is beyond my tolerance, I will feel especially tired.”

## Discussion

Our literature review provides insight into the factors influencing English teachers’ emotional labor in previous studies. English Teachers have excessive workloads, dealing with students who were silent, had mental problems or were xenophobic (Taylor, 2020), research expectations (Hattie and Marsh, 1996), demanding students, ambitious leaders (Chan, 2006), high professional standards (Leithwood and Beatty, 2008), these make their work very stressful and emotionally draining. All of this is relevant to the study of teacher emotions, but we need to consider that in the context of the COVID-19, where online teaching has become a common phenomenon in teaching and learning. Emotional experiences are psychological, interactional, and social processes (Denzin, 2007), computer-mediated elements in online teaching are embedded in this interactive process as part of the English teacher’s work (Atmojo and Nugroho, 2020). Therefore, we explored the emotional labor of English teachers from the perspective of online teaching. We found that the factors influencing Chinese English teachers’ emotional labor in the context of online teaching were different from those discussed in previous studies based on the reality space. Previous studies have looked at the social relations in which teachers’ identities are located to discuss the factors that influence their emotional labor, and as some researchers have said, when identities activate emotions, their motivational mechanisms are linked to the diverse networks that individuals have and the expansion of these networks (Stryker and Serpe, 1982). At the macro level, the factors influencing English teachers’ emotional labor are linked to language, society, and culture, and at the micro level, a variety of factors such as teacher-student interactions, teacher-administrator relationships, and individual teacher characteristics are examined. But the online teaching process, in which the computer network is embedded as a technological factor in the daily work of English teachers, breaks the original teaching procedures and becomes a new kind of interactive relationship. We found that there are three main factors that influence Chinese English teachers’ emotional labor in online teaching: the degree of adaptation to online teaching technology, the invisibility of the online teaching space, and the peripheral environment of the space in which the teachers were located. We considered computer-mediated teaching as a new interactive relationship, analyzed Chinese English teachers’ emotional labor, and thus were not the same influencing factors as those revealed in previous papers. In addition, we conclude that the first two influences are at a priority level for Chinese English teachers, while whether the third factor can bring positive or negative emotions is often influenced by the nature of the emotions brought by the first two factors. We examined this phenomenon using normative theory, where the first two factors are in a strong link with norms and are artificially direct factors, while the third factor is in a weak link with norms and is accidentally indirect. As our interviewees talked about, the noise brought from the external environment is a norm that they perceive themselves because, after all, it affects teaching and learning, but there is nothing in the school management norms that does not allow such contingencies. We see that if the first two influences bring positive emotions, then English teachers will be more likely to respond positively to the effects of the third factor, and vice versa they will tend to respond negatively. Thus, we also have a reflection on the normative theory, where norms have been considered as guidelines that bind individual teachers or groups of teachers, but conversely, emotions can influence English teachers’ perceptions of norms. When English teachers were sustained by positive emotions brought about by factors strongly associated with norms, they would reduce negative emotions brought about by factors weakly associated with norms. And conversely, when they were sustained by negative emotions brought about by factors strongly associated with norms, they would enhance negative emotions brought about by factors weakly associated with norms.

We have found in previous studies that when discussing dilemmas, most researchers have discussed the negative effects that English teachers face after emotional labor (which we have written about in our literature review, such as leaving the profession at high rates, etc.). But our study found that English teachers in the labor process also face some dilemmas in terms of emotional choices and behaviors. We need to emphasize the point that emotional labor is a process that has a time span and is also divided into different steps, being stimulated by an event before emotional labor, making some appropriate emotional choices, and presenting behaviors during emotional labor, and the effects after emotional labor. Previous studies will focus on the negative effects after emotional labor, which is a dilemma English teachers may face, but we will focus on the process of conducting emotional labor, and we examined the situation in the context of online teaching, explored what kind of emotional labor dilemmas Chinese English teachers face in this process. It is the change in influencing factors that leads to these emotional labor dilemmas. When they are stimulated by an event to produce negative emotions, they need to evaluate and choose appropriate emotions in relation to their own identity, social norms, and social expectations, considering various reasons such as teaching norms, teaching efficiency, and students’ psychology, and in this process, they face three main dilemmas: the emotional rule dilemma, the emotional expression dilemma, and the professional identity dilemma. In detail, English teachers are influenced by the culture of their linguistic counterparts and the emotional rules that come with online teaching technology, so perceive emotional rules somewhat differently; When English teachers encounter bad behavior of students in classroom management should they have emotional words or expressions; English teachers often feel beyond what teachers should do in their choice of emotions and behaviors and thus doubt the boundaries of their own identity. Although normative theory suggests that the existence of norms compels teachers to follow professional norms and regulatory rules, we need to see that normative theory is in terms of social norms, while emotions possess not only social but also biological properties. That is, it acts on people’s social identities, but the biological nature of emotions is not limited by normative theory, and thus normative theory has limited explanatory power for emotional labor. Teachers as social identities are framed by several social norms in their behavior, but teachers as selves, the biological nature of emotions will be outside of social norms and they tend to turn to their self-feelings without considering social norms. Thus, during the emotional labor of English teachers teaching online, they face a conflict between social norms and self-feelings, which can also be described as a conflict between the social and biological nature of emotions.

Some previous studies have been devoted to explaining the strategies of English teachers’ emotional labor (Lee and Van Vlack, 2018; Pervaiz et al., 2019; Taylor, 2020). When we try to explain these strategies, we first need to clarify why they emerge, that is, what are the factors and dilemmas that influence the emotional labor of English teachers teaching online. In this article, we did not go to focus our study on the emotional labor strategies of English teachers teaching online, but the issue is equally worthy of study and we provide a prior study for the exploration of this issue.

We believe that the strategy of emotion management is only to let the unpleasantness we feel put aside temporarily so that emotions do not interfere with normal teaching during work hours, but not to make the emotional reaction disappear. Our interviewees told us that they still face the situation of dealing with negative emotions outside of work time, such as confiding in their families, doing exercise, eating some good food, etc. We should not ignore the relationship between emotions and memories, which are stored in our memories and evoked in similar situations and the physical discomfort caused by negative emotions is still existent. In conclusion, the factors influencing the emotional labor of English teachers teaching online is the negotiation between norms and emotions, where teachers ensure that teaching and learning are carried out properly by dealing with an element first, without making negative emotions or physical reactions disappear, only hiding them temporarily. During long hours of work, emotions as having physiological properties need to be released in time, and they sometimes do not know how their feelings and emotions need to be suppressed, because emotions are inherent physiological patterns of human beings, which are evolved adaptive responses (Panksepp, 2005).

Compared to previous studies, our examination of the factors influencing Chinese English teachers’ online teaching emotional labor and the emotional labor dilemmas they face has both theoretical and practical implications. Theories related to emotional labor suggest that people in a work situation must follow some rules of emotional labor, but through this study, we see resistance in the emotional labor process. Our attempt to add new online contexts to the emotional labor theory will also show more researchers what factors influence the emotional labor of language teachers teaching online. As I wrote in the literature review, previous studies have tended to examine the influencing factors of English teachers’ emotional labor by focusing more on individual aspects of English teachers’ characteristics, but we argue through this study that the influencing factors of English classroom emotional labor also include factors other than individuals, because online teaching adds the Internet as an important mediator. Most importantly, the English teacher’s object of interaction has changed: from facing students to facing computers. This change brought a new mechanism of action for the emotional labor of English teachers. We attempt to analyze these influences to bring more researchers’ attention to the emotional labor of language teachers teaching online and to bring further research to this academic topic.

Furthermore, the emotional labor of English language teachers is not a new topic, but the emotional labor of English language teachers teaching online is a new topic. The influencing factors of English teachers’ emotional labor in online teaching need to be paid attention, because it raises new dilemmas about English language teachers’ emotional labor. We also need to note that many studies that talk about English teachers’ dilemmas tend to talk about what negative effects follow English teachers’ emotional labor, without exploring what difficulties and dilemmas English teachers face when they engage in emotional labor. This is the significance of this study in terms of practice: to promote the alleviation of English language teachers’ emotional labor dilemmas.

## Conclusion and suggestion

We examined two questions: What factors influence Chinese English teachers’ emotional labor in online teaching? What dilemmas Chinese English teachers face in their emotional labor in online teaching? There are many studies that have examined the factors that influence the emotional labor of teachers, especially English language teachers, but this is all in the context of teaching in a real space, and we need to be aware of what influences the emotional labor of English language teachers teaching online. We found through interviews with Chinese English teachers that the factors influencing the emotional labor of English teachers teaching online include three main aspects: the degree of adaptation to online teaching technology, the invisibility of the online teaching space, and the peripheral environment of the space in which the teachers were located. In addition, these influencing factors lead to emotional labor dilemmas. We concluded that they often faced three dilemmas of emotional labor when teaching online: emotional rule dilemma, emotional expression dilemma, and professional identity dilemma.

In the existing studies, there are relatively more studies on language teachers’ emotions. But in the context of the COVID-19, online teaching has become a common way in the teaching process and teachers’ emotional labor has taken on new contexts and occasions. The difference being that teachers face more interpersonal relationships in offline teaching and more human-machine relationships in online teaching, so we explore the factors of online teaching that affect Chinese English teachers’ emotional labor in online teaching, and on this basis analyze the emotional labor dilemmas they face, and finally propose some suggestions to solve the dilemmas. This paper attempts to provide a new perspective for future research on teachers’ emotions, calls for researchers to pay attention to emotional labor in the process of online teaching, and provides inspiration for how to enhance English teachers’ well-being. The paper also corrects some ideas of the original study, such as teachers’ use of emotional strategies can only hide negative emotions, negotiate with negative emotions, and choose to prioritize the assessed results, but not make negative emotions go away. In addition, we offer some reflections on normative theory. An examination of the factors influencing English teachers’ emotional labor for online teaching revealed that although norms can make English teachers comply with multiple social norms and regulations, English teachers’ emotions can influence their perceptions of norms. An analysis of the dilemmas of English teachers’ online teaching emotional labor revealed that normative theory has limited explanatory power for English teachers’ online emotional labor because it is some framework for social identity and does not consider the biological nature of emotions. Meanwhile, this paper also provides some space for future research, such as gender differences in online teaching emotional labor and variations in online teaching emotional labor strategies, which are worthy of further research.

However, there are limitations to our study. In our paper, we only examined the influencing factors of English teachers’ emotional labor for online teaching and the dilemmas they faced during their emotional labor, but we did not observe and analyze their emotional labor strategies for online teaching. Emotional labor strategies, which are the behavioral outcomes of English teachers’ ultimate emotional choices in the face of dilemmas, are also highly related to the influencing factors, but we did not further examine English teachers’ emotional strategies due to our energy and choice of research focus, which affects the paper’s overall presentation of Chinese English teachers’ emotional labor in online teaching. In addition, our study may have been affected by the limited number of interviewees, even though gender and age were considered in the interviewees.

There is no doubt that the emotional labor dilemma will lead to a decrease in teachers’ creativity and happiness, and we try to give some suggestions to alleviate the dilemma. (1) English teachers need to improve their emotional competence. Being emotionally competent has tacitly become a prerequisite for the teaching profession (Korotaj and Mrnjaus, 2021). Although teachers cannot make the feelings and physical reactions brought by negative emotions disappear, we can change the conditions under which emotions occur and reduce the possibility of negative emotions or the harm brought by negative emotions by improving emotional competence. (2) Improve the working environment of teachers. A negative work environment can cause physical, psychological, and occupational problems (Martínez et al., 2020). This requires school administrators to strengthen their care for teachers, understand the difficulties they encounter at work, and empathize with them. (3) Improve literacy in the use of educational software. This requires schools to provide guidance to teachers and make timely explanations about the functionality of the software, so that teachers’ time is maximized in the teaching process rather than in solving technical problems, which also improves teachers’ work efficiency and likewise reduces the time spent sitting in front of the computer, thereby improving teachers’ physical and mental health.

## Data availability statement

The original contributions presented in the study are included in the article/supplementary material, further inquiries can be directed to the corresponding author.

## Author contributions

HW read the literature, designed the study, contacted interviewees, observed the online teaching, and compiled the interview data. NS participated in the interviews and observations, analyzed some literature, and made some useful suggestions for teacher emotion research. All authors contributed to the article and approved the submitted version.

## Conflict of interest

The authors declare that the research was conducted in the absence of any commercial or financial relationships that could be construed as a potential conflict of interest.

## Publisher’s note

All claims expressed in this article are solely those of the authors and do not necessarily represent those of their affiliated organizations, or those of the publisher, the editors and the reviewers. Any product that may be evaluated in this article, or claim that may be made by its manufacturer, is not guaranteed or endorsed by the publisher.
